# Small-angle scattering tensor tomography algorithm for robust reconstruction of complex textures

**DOI:** 10.1107/S205327332300863X

**Published:** 2023-10-19

**Authors:** Leonard C. Nielsen, Paul Erhart, Manuel Guizar-Sicairos, Marianne Liebi

**Affiliations:** a Department of Physics, Chalmers University of Technology, Gothenburg, Sweden; b Paul Scherrer Institute (PSI), Villigen, Switzerland; c École Polytechnique Fédérale de Lausanne (EPFL), Lausanne, Switzerland; Institute of Crystallography - CNR, Bari, Italy

**Keywords:** tensor tomography, SAXS, small-angle X-ray scattering, scattering, reciprocal-space maps, optimization

## Abstract

The development of small-angle scattering tensor tomography has enabled the study of anisotropic nanostructures in a volume-resolved manner. It is of great value to have reconstruction methods that can handle many different nanostructural symmetries. For such a method to be employed by researchers from a wide range of backgrounds, it is crucial that its reliance on prior knowledge about the system is minimized, and that it is robust under various conditions. Here, a method is presented that employs band-limited spherical functions to enable the reconstruction of reciprocal-space maps of a wide variety of nanostructures. This method has been thoroughly tested and compared with existing methods in its ability to retrieve known reciprocal-space maps, as well as its robustness to changes in initial conditions, using both simulations and experimental data. It has also been evaluated for its computational performance. The anchoring of this method in a framework of integral geometry and linear algebra highlights its possibilities and limitations.

## Abstract 2

The development of small-angle scattering tensor tomography has enabled the study of anisotropic nanostructures in a volume-resolved manner. The paper presents a method tested against both simulations and experimental data, and compared with existing methods, demonstrating its ability to handle several different classes of nanostructures.

## 1. Introduction

Small-angle X-ray scattering (SAXS) probes the nanometre-scale variations in the electron density of materials averaged over areas typically in the range of 1 × 1 µm to 1000 × 1000 µm, depending on the size of the X-ray beam. The data from a SAXS experiment carry information about the nanostructure of a sample, including characteristic length scales and orientation, and have been used to study numerous materials (Fratzl *et al.*, 1996 ▸; Georgiadis *et al.*, 2016 ▸; Lichtenegger *et al.*, 1999 ▸). Scanning SAXS can be performed across a sample to yield a two-dimensional map, with each scanned pixel associated with a corresponding two-dimensional cut through the reciprocal-space map (Fratzl *et al.*, 1997 ▸; Pabisch *et al.*, 2013 ▸; Paris, 2008 ▸). Since a SAXS measurement with an area detector gives two-dimensional data, measurements must be performed at several angles to obtain three-dimensional data from a sample (Liu *et al.*, 2010 ▸; Seidel *et al.*, 2012 ▸; Georgiadis *et al.*, 2016 ▸). By rotating a three-dimensional sample around a single axis, standard tomographic reconstruction can be used in cases where the sample scattering is isotropic or when the scattering is symmetric about the axis of rotation (Stribeck *et al.*, 2006 ▸, 2008 ▸; Feldkamp *et al.*, 2009 ▸; Schroer *et al.*, 2006 ▸; Álvarez-Murga *et al.*, 2012 ▸; Jensen *et al.*, 2011 ▸).

With the use of a tilt angle in addition to rotation, recent works by Schaff *et al.* (2015 ▸), Liebi *et al.* (2015 ▸, 2018 ▸) and Gao *et al.* (2019 ▸) present tomographic methods for the reconstruction of the three-dimensional reciprocal-space map using scanning SAXS projections, or small-angle X-ray scattering tensor tomography (SAXSTT).

Whereas Schaff *et al.* (2015 ▸) demonstrated a two-step procedure of first reconstructing the reciprocal-space map without any data-reducing assumptions and then analyzing this reconstruction to find orientations, the approaches of Liebi *et al.* (2015 ▸, 2018 ▸) (referred to as the zonal harmonics or ZH method) and Gao *et al.* (2019 ▸) (referred to as the iterative reconstruction or IR method) use reduced models of the reciprocal-space maps. The ZH method of Liebi *et al.* (2015 ▸, 2018 ▸) models the reciprocal-space map of each voxel using squared band-limited spherical functions expressed in spherical harmonics. The demonstrations of the method by Liebi *et al.* (2015 ▸, 2018 ▸) used a reduced model, employing only zonal harmonics (spherical harmonics symmetric about an axis of rotation) and two Euler angles, with the angles parameterizing the main nanostructural orientation. The IR method of Gao *et al.* (2019 ▸) uses the symmetric rank-2 tensor as the basis for its model. The work of Gao *et al.* (2019 ▸) primarily describes the reconstruction of a proposed orientation distribution function derived heuristically for fiber scattering. The proposed orientation distribution function approach is not compatible with reciprocal space map based models of SAXSTT, because it does not yield the necessary invariance with respect to rotation of the sample (*e.g.* De Falco *et al.*, 2021 ▸). However, as noted in a footnote by Gao *et al.* (2019 ▸), it is also possible to configure the IR algorithm to reconstruct the reciprocal-space map directly, which is the way IR was employed in this work.

The use of a complete band-limited basis of even-ordered spherical harmonics described by Liebi *et al.* (2018 ▸) has to the best of our knowledge not been implemented or tested for SAXSTT as of the writing of this work. Moreover, the approach of Liebi *et al.* (2015 ▸, 2018 ▸) requires fitting the measured reciprocal-space map to sums of squared polynomials, which is a difficult class of optimization problems to solve (Ahmadi *et al.*, 2017 ▸), as it results in a non-linear system of equations. Here, we present spherical integral geometric tensor tomography (SIGTT) as an improvement on the complete basis approach proposed by Liebi *et al.* (2015 ▸, 2018 ▸). SIGTT eliminates the squaring of the polynomial which was used in the ZH approach, resulting in a linear system, and its implementation does not rely on Euler angles.

## 2. Results

### 2.1. Theory

The equation for the measured reciprocal-space map (RSM) of SAXS may be written as 

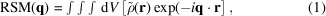

where 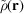 is the auto-correlation function of the electron density of the probed volume, **r** is the position and **q** is the reciprocal-space vector. Consider this integral over a region of space (a voxel) and at a fixed ||**q**||. Then, 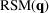 reduces to 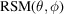 , a function on the unit sphere, which may by theorem be represented by an infinite series of spherical harmonics (Kosmann-Schwarzbach & Singer, 2010 ▸). As discussed in previous work by Liebi *et al.* (2018 ▸), the summation is reduced to the even orders as a result of Friedel symmetry. The measured reciprocal-space map at a single **q** and at a particular position **r** in space may then be written 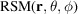 and expanded in spherical harmonics as 

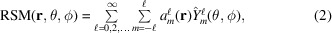

where θ and ϕ are, respectively, the polar and azimuthal angles of the reciprocal-space map, 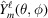 is the real-valued spherical harmonic basis function of order ℓ and degree *m*, and 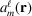 is the coefficient of that basis function at position **r**. Note that the summation over ℓ in equation (2) only includes even terms, as indicated by the subscript of the summation sign.

The following projection model is based on the discrete model of Liebi *et al.* (2018 ▸), cast in terms of line integrals within the sample coordinate system, which map it onto a projection space. The projection space is spanned by four coordinates (*j*, *k*, α, β), which can be mapped to experimental parameters. The linear coordinates, (*j*, *k*), map to the vertical and horizontal positioning of the sample during scanning SAXS. The angular coordinates (α, β) map to rotations of the sample about laboratory axes orthogonal to the beam direction during the measurement. Rotations about axes which are not orthogonal to the beam direction can be handled by decomposition into beam-orthogonal rotations that map to (α, β), and beam-parallel rotations that map to transforms of (*j*, *k*). The projection of a scalar function *f*(**r**) from three to two dimensions at an arbitrary angle for a narrow beam is described by the John transform, also known as the X-ray transform, which may be expressed 

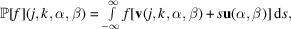

where α and β are the azimuthal and polar angles, respectively, with respect to a fixed plane in the sample coordinate system, for a line of integration which intersects with the system’s origin. Then, *j* and *k* give the line’s offset from the origin in the plane of projection, which is orthogonal to the line’s direction. In this representation, parameterized by *s*, **v** gives the position of the beam in the plane of projection, whereas **u** gives its direction. See Note 1 in the supporting information for more details on how to map the sample coordinate system to an experimental coordinate system.

To keep our equations compact, we will use the shorthand notation 

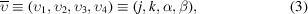

such that 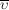 represents simultaneously a line of integration in three-dimensional space and a measured point in projection space. By inserting equation (2) into the John transform 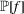 , we obtain an expression for the projection of the spherical harmonics from three to two dimensions using two projection angles: 

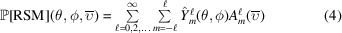

with 

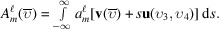




From equation (4) it is apparent that both the reciprocal-space map at a particular point in space and the projection of it may be represented using an even-ordered spherical harmonic expansion with a one-to-one correspondence between the representations. In other words, each order and degree in the projected harmonic can be regarded as the projection of a single order and degree in the harmonics distributed in space. However, small-angle scattering does not permit us to probe the entirety of the reciprocal-space map at a single projection angle; instead, it probes a set of points which lie approximately on a great circle spanned by the set of unit vectors orthogonal to the direction of the beam. We describe this using a parametric curve *C*(φ, α, β), where for fixed α and β, *C*(φ) is a great circle orthogonal to the direction of **u**.

We can then write the model (which we will denote 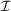 ) for a single measured reciprocal-space map as 

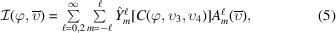

which completes the forward model of SIGTT. Anisotropic scattering signals have previously been modeled using line integrals of spherical polynomials by Wieczorek *et al.* (2016 ▸) for dark-field tomography. The key difference of SIGTT from the spherical harmonic model described by Liebi *et al.* (2018 ▸) is the linearity of the system, which is achieved by not squaring the spherical harmonics. Unlike the implementation demonstrated by Liebi *et al.* (2015 ▸, 2018 ▸), SIGTT does not employ a local coordinate system for the reciprocal-space map in each voxel.

These changes result in the preservation of the orthogonality of each component of the per-voxel model, and simplify gradient calculations. See Note 2 in the supporting information for how the SIGTT representation maps to Cartesian tensors, as in the model used by IR. The inverse problem of obtaining 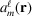 is solved by using a regularized least-squares approach (Section 4).

### 2.2. Simulations

**Figure 1:**
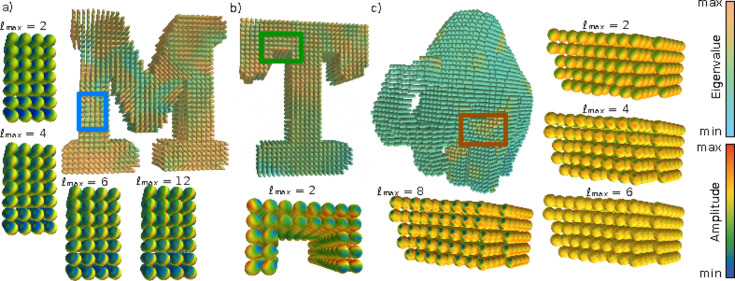
Superquadric glyph render of simulations. (*a*) Sample ‘M’ with its zonally symmetric reciprocal-space maps from one region (blue square), truncated at 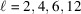 . (*b*) Sample ‘T’ with its rank-2 tensor reciprocal-space maps from one region (green square). (*c*) Sample ‘mammoth’ with its unrestricted ℓ_max_ = 8 reciprocal-space maps from one region (orange square), truncated at 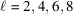 . The colors of the superquadric glyph renders show the largest eigenvalue of the rank-2 tensor component of each simulated sample, and the upper and lower bounds of the color mapping are individually set to reveal the texture of each sample. The colors of the reciprocal-space map renders show their amplitudes and are scaled to the maximum and minimum amplitude in the selected region of each sample.

In order to compare and evaluate the different methods, a simulation framework was developed (Section 4). In total, three simulations were created, labeled ‘M’, ‘T’ and ‘mammoth’; see Fig. 1 ▸ for an overview. Sample ‘M’ is intended to provide a simulated reciprocal-space map with an intensity distribution that possesses the zonal symmetry assumed by ZH, meaning that the distribution has an axis of rotational symmetry. This is done by constraining its simulated reciprocal-space map to be approximately described by zonal harmonics (which are defined by an expansion of spherical harmonics with *m* = 0 and an axis of symmetry) up to ℓ_max_ = 12. In the simulation of ‘T’, the reciprocal-space map is described entirely by symmetric rank-2 tensors, which is the model employed by IR. Finally, the simulation of ‘mammoth’ employed spherical harmonics up to ℓ_max_ = 8, with a weak ℓ = 2 component, to model reciprocal-space maps with more complicated symmetries. The reconstructions of each method are compared with the simulated samples by calculating the squared Pearson correlation coefficient *R* ^2^ [equation (15)] of the simulated and reconstructed reciprocal-space maps on a voxel-by-voxel basis.

**Figure 2:**
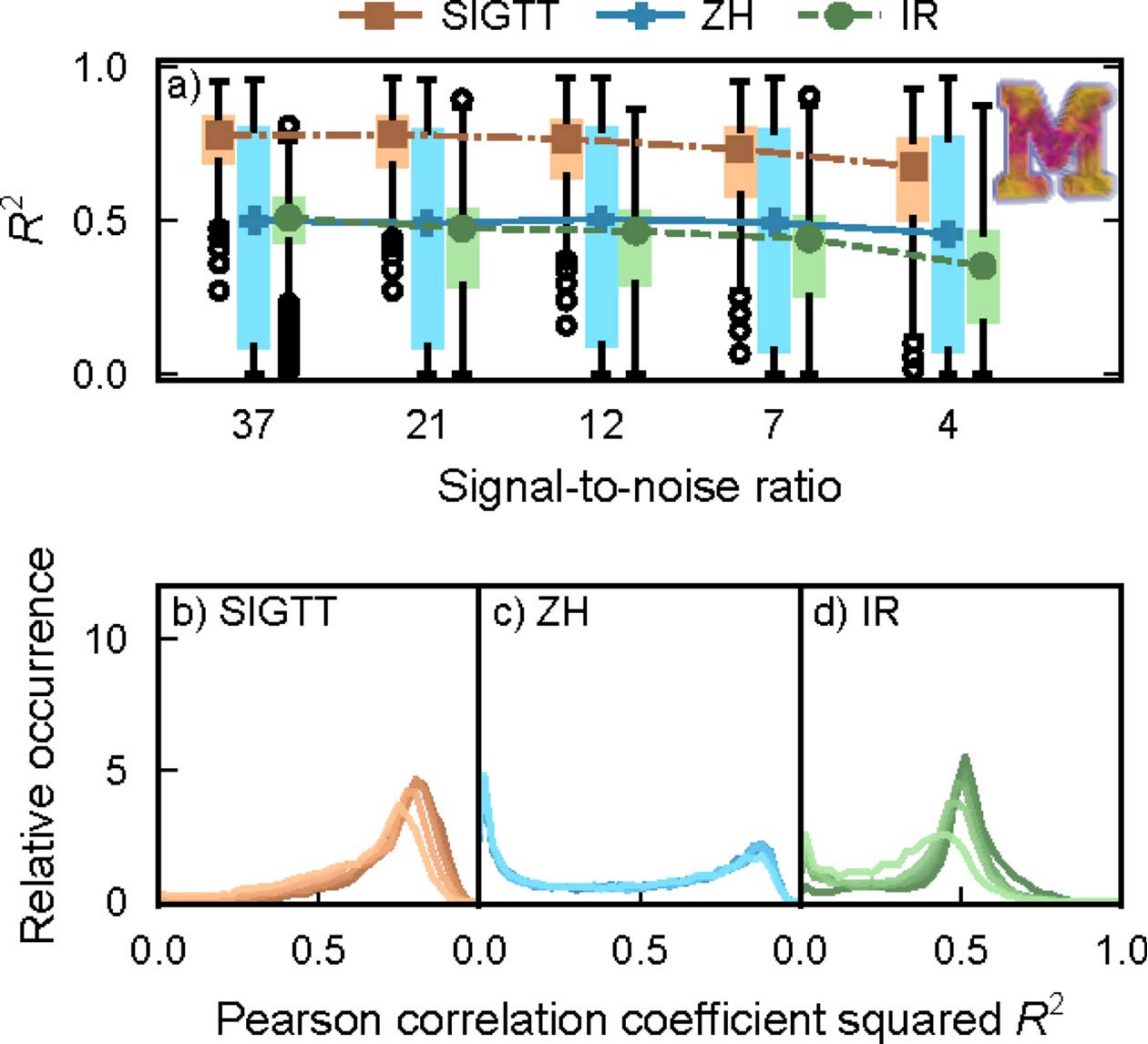
Correlations for sample ‘M’. (*a*) Box plots of *R* ^2^ as defined in equation (15), with lines and symbols indicating the respective median of each box plot. Outlier dots each represent 100 voxels. (*b*)–(*d*) Correlation coefficient distribution for each of the three methods. The signal-to-noise ratio goes from 37 (darkest lines) down to 4 (lightest lines). The image inset shows a volume render of the simulated sample.

In Fig. 2 ▸, the results from reconstructing simulated data for sample ‘M’, which possesses an intensity distribution with zonal symmetry, are shown. See Section 4 for details on the box plots. SIGTT performs the best in the comparison, with a peak correlation centered around *R* ^2^ = 0.8 at the highest signal-to-noise ratio (SNR), decaying down to about 0.75 at the lowest SNR, with a greater interquartile range. The best possible performance of SIGTT in this comparison is constrained by the fact that the chosen discretization of the reciprocal-space map permits the fitting of harmonics only up to ℓ = 6, whereas the sample contains harmonics up to ℓ = 12. The reconstruction is also affected by the missing wedge problem, a common limitation in tomography when only a subset of projection space is sampled. The performance of ZH is almost unaffected by the SNR, with its correlation centered around *R* ^2^ = 0.5 with a large interquartile range. In the lower middle plot, a peak can be seen both around 0.8 and around 0, suggesting a mixture of good and poor performance across the sample volume. The trend in the performance of IR is more similar to that of SIGTT in that its correlation decays and its interquartile range increases with the decrease in SNR. However, its maximum possible correlation to most of the sample is bounded at around 0.5 by the fact that it can only correlate to the ℓ = 2 component of the model, due to being restricted to the symmetric rank-2 tensor.

**Figure 3:**
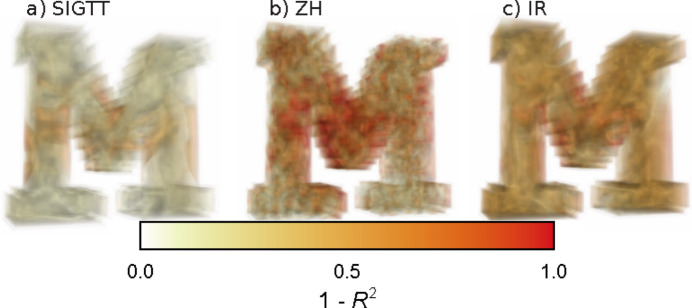
Volume renders of errors for reconstructions of ‘M’. (*a*) Errors for SIGTT. (*b*) Errors for ZH. (*c*) Errors for IR. The error is defined as 1 − *R* ^2^, where *R* ^2^ is given by equation (15). Larger errors are rendered with greater opacity and are thus visible even if they are in the interior.

Volume renders of the errors of each method for ‘M’ can be seen in Fig. 3 ▸. It is clear from the figure that all methods have larger errors in the middle regions of the model, but that these are larger for ZH and IR. Moreover, outside these regions, the error for SIGTT is closer to 0.

**Figure 4:**
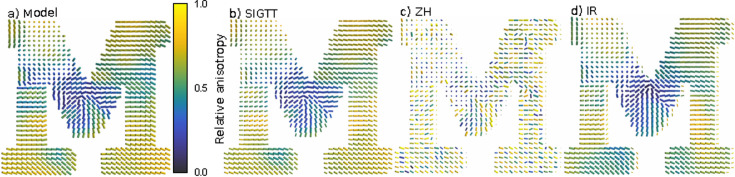
Comparison of virtual slices of ‘M’. (*a*) Virtual slice of simulated sample ‘M’. (*b*) SIGTT reconstruction. (*c*) ZH reconstruction. (*d*) IR reconstruction. The glyphs are colored according to the relative anisotropy [equation (14)] and scaled according to the isotropic component [equation (13)] of each reciprocal-space map. All three reconstructions follow the orientations of the model reasonably well on average. The SIGTT reconstruction follows both the orientations and relative anisotropy of the model closely. The ZH reconstruction has a lot of variation in the relative anisotropy, as well as many orientations deviating from the overall tendency to follow the model. The IR reconstruction follows the model almost as well as the SIGTT reconstruction.

In Fig. 4 ▸ are virtual slices with glyphs showing the orientation of each reciprocal-space map for (*a*) the simulation ‘M’, (*b*) SIGTT, (*c*) ZH and (*d*) IR. See Note 3 (supporting information) for details on the orientation analysis. The comparison shows that the orientation of the reciprocal-space maps in the simulation is overall reasonably well followed by all reconstructions. However, from (*c*) it is clear that the ZH reconstruction contains many deviations from the simulated orientation. In terms of the relative anisotropy [equation (14)], indicated by the color of each glyph, it is generally well followed by SIGTT, as seen in (*b*), but poorly followed by ZH, seen in (*c*). It can be inferred from this that the numerous deviations in the ZH reconstruction are the cause of the large dispersion in *R* ^2^ seen in Fig. 2 ▸(*a*). IR, in (*d*), gives a reconstruction that follows the orientations and relative anisotropy nearly as well as (*b*), with the exception of certain regions. This is to be expected, since while the anisotropy in fiber-like symmetry is generally dominated by the rank-2 tensor component, this will not be the case everywhere.

**Figure 5:**
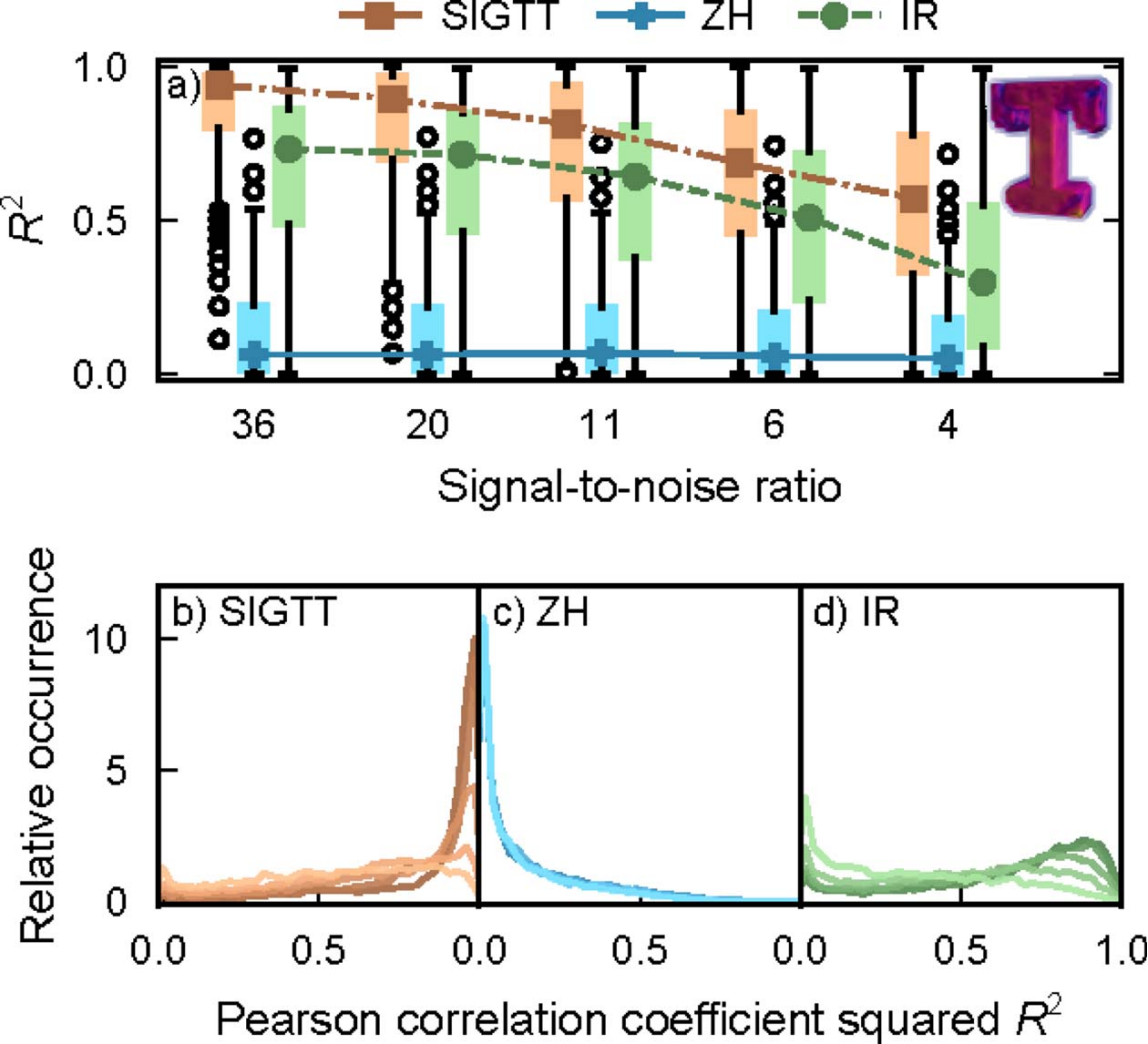
Correlations for sample ‘T’. (*a*) Box plots of *R* ^2^ as defined in equation (15), with lines and symbols indicating the respective median of each box plot. Outlier dots each represent 100 voxels. (*b*)–(*d*) Correlation coefficient distribution for each of the three methods. The signal-to-noise ratio goes from 36 (darkest lines) down to 4 (lightest lines). The image inset shows a volume render of the simulated sample.

In Fig. 5 ▸ the performance between the three models is compared in reconstructing sample ‘T’, which consists of reciprocal-space maps with ℓ_max_ = 2. SIGTT and IR perform fairly similarly, with the performance of IR consistently being somewhat worse. Since these models are very similar for the special case of ℓ_max_ = 2, this difference in performance is likely related to the fact that the IR implementation uses a less precise projection algorithm, as well as its use of fixed step size steepest descent gradient optimization. It is likely the case that SIGTT performs better due to modeling the measured reciprocal-space map as a line integral on the reciprocal-space sphere, rather than as a point, as well as using continuity-enforcing regularization. ZH performs very poorly regardless of noise level in the case of ‘T’. This is likely both because it cannot directly represent coefficients of the ℓ = 2 harmonics due to its use of squared polynomials, and because ‘T’ does not follow zonal symmetry, meaning it does not have an axis of rotational symmetry, and cannot be represented by a rotated spherical harmonic expansion with only *m* = 0 components.

**Figure 6:**
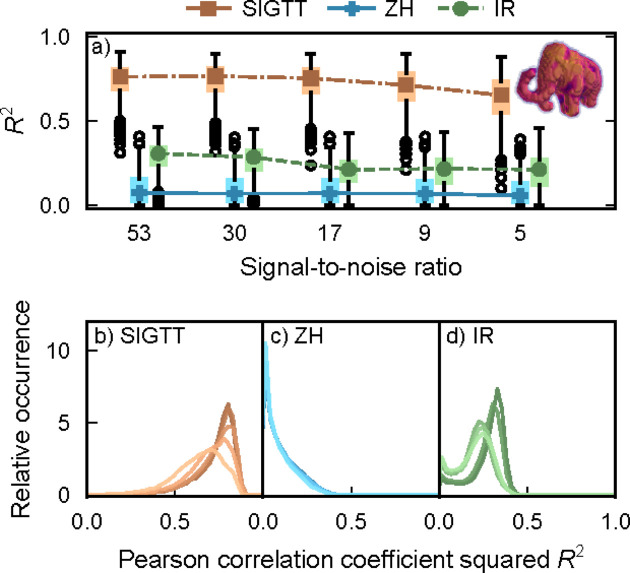
Correlations for sample ‘mammoth’. (*a*) Box plots of *R* ^2^ as defined in equation (15), with lines and symbols indicating the respective median of each box plot. Outlier dots each represent 100 voxels. (*b*)–(*d*) Correlation coefficient distribution for each of the three methods. The signal-to-noise ratio goes from 53 (darkest lines) down to 5 (lightest lines). The image inset shows a volume render of the simulated sample.

The results from the reconstruction of the sample ‘mammoth’ are seen in Fig. 6 ▸. SIGTT has similar performance to the case of sample ‘M’, with its correlation starting around 0.8 and decaying to about 0.65 as the SNR decreases, with an increase in the interquartile range. ZH performs very poorly, which is to be expected, as the ‘mammoth’ sample does not follow zonal symmetry at all. IR performs better than ZH, but is bounded by the fact that the reciprocal-space maps in this simulation only have a weak rank-2 tensor component. Thus, it does not reach a median *R* ^2^ above 0.3. See Note 4 and Figs. S1 and S2 (in the supporting information) for volume renders of the errors of ‘T’ and ‘mammoth’, as well as Fig. S3 and equation (S1) for a comparison of the anisotropic power distribution of ‘mammoth’ and ‘M’.

**Figure 7:**
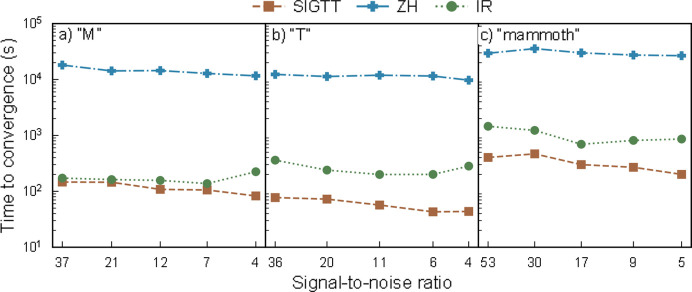
Timing comparison of methods. (*a*) Timing for sample ‘M’. (*b*) Timing for sample ‘T’. (*c*) Timing for sample ‘mammoth’. Note the logarithmic *y* axis. In all cases, SIGTT is the fastest method, followed by IR, with ZH being the slowest by a considerable margin.

In Fig. 7 ▸ we show timing information for the reconstructions of (*a*) ‘M’, (*b*) ‘T’ and (*c*) ‘mammoth’. Note the logarithmic *y* scale. In all cases, SIGTT is the fastest method, followed by IR, with ZH being more than an order of magnitude slower than either method. For example, one of the slowest cases for all methods is ‘mammoth’ with SNR 30 [panel (*c*)] – in this case, SIGTT requires approximately 7 min, IR requires approximately 25 min (3.5 times as long as SIGTT) and ZH requires approximately 10 h (85 times as long as SIGTT). In the case of ‘M’ [panel (*a*)], IR is nearly as fast as SIGTT, although it should be noted that in this case SIGTT is fitting 28 coefficients, whereas IR is fitting six coefficients. ZH is slower than both methods by approximately two orders of magnitude. While ZH also only uses six degrees of freedom in this case, two of these degrees of freedom are polar and azimuthal angles, and it models the reciprocal-space map using the squared amplitude of spherical functions. Both of these features lead to an optimization problem which is more difficult to solve due to its non-linearity (*e.g.* Hochbaum, 2007 ▸; Ahmadi *et al.*, 2017 ▸). For ‘T’ [panel (*b*)], both SIGTT and IR are fitting six coefficients, as ‘T’ has only rank-2 tensor components, and in this case SIGTT is several times faster. The sample ‘mammoth’ [panel (*c*)] takes substantially longer to fit for all of the methods, as it has a greater volume (60 × 60 × 80 voxels, whereas ‘M’ and ‘T’ have 50 × 50 × 50 voxels each), increasing the amount of time it takes to compute each projection. SIGTT is several times faster than IR here, and approximately two orders of magnitude faster than ZH. This comparison should only be understood as a broad guideline to the performance of the methods; each implementation has a number of parameters which affect convergence in different ways, which were adjusted with the aim of obtaining a reconstruction that correlates well with the simulation, rather than optimized for speed. Moreover, SIGTT is implemented in Python, whereas ZH and IR are implemented in Matlab, which means that the conditions for optimizations such as multithreading and efficient memory handling are different (see Section 4.6). It is therefore likely that at least some of the speedup seen when comparing SIGTT with the other methods is due to a more efficient implementation, *i.e.* the particular code used to carry out computations, rather than improvements in the basic method. It is likely that all methods could be sped up considerably by employing a GPU-based projection algorithm (*e.g.* Nikitin, 2023 ▸).

### 2.3. Experimental data

**Figure 8:**
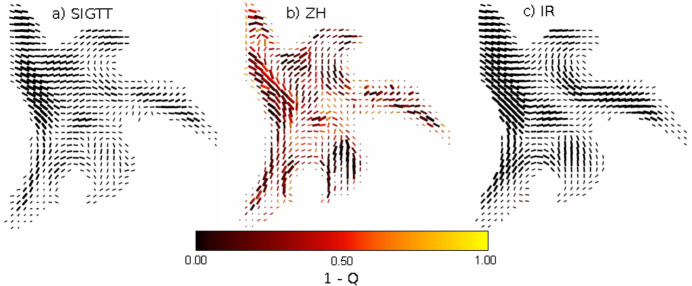
Experimental ensemble reconstructions. (*a*) Virtual slice from an ensemble of ten reconstructions each with randomized initial conditions of a sample of trabecular bone using SIGTT. (*b*) Ensemble reconstruction using ZH. (*c*) Ensemble reconstruction using IR. The glyphs are scaled by the square root of the anisotropic power, defined in equation (11). The quantity *Q*, defined in equation (16), is a measure of how much the anisotropy of each reciprocal-space map changes across the ensemble of reconstructions. The methods generally agree in terms of the orientation of each reciprocal-space map, but only ZH shows a change in the anisotropy across the ensemble.

An ensemble of ten reconstructions, each with some initial conditions randomized, were performed using SIGTT, ZH and IR on a sample of trabecular bone. For experimental details, see Liebi *et al.* (2015 ▸), sample B. The chosen *q* range of 0.0379–0.0758 nm^−1^ for this reconstruction does not contain the collagen meridional peak, and therefore its reciprocal-space map has fiber symmetry from equatorial diffuse mineral scattering. A comparison of a virtual section from each of the three methods can be seen in Fig. 8 ▸. Because there is no ground truth with which to compare for experimental data, the ensemble of reconstructions was instead analyzed to investigate the robustness of each method against perturbations in the initial conditions. The spherical harmonic representations of the ten reconstructions were averaged over, voxel-by-voxel, and Fig. 8 ▸ shows the results of the averaged reconstruction. The colors of the orientation glyphs indicate the degree to which the anisotropy of the reciprocal-space map changes across every reconstruction; the quantity *Q* is defined in equation (16). The glyphs are scaled according to the square root of the mean anisotropic power of each reciprocal-space map [the anisotropic power is defined in equation (11)] across the ensemble. The results indicate that SIGTT and IR are robust to perturbations of the initial conditions, but that ZH is not. However, the orientations of the averaged ZH reconstruction agree well with those of SIGTT and IR. A plausible reason for this difference is that ZH is the only method out of the three which depends on Euler angles. Depending on the initial conditions of the angles, the solution may be confined to local minima, as the symmetries of its reciprocal space can only vary across a limited subspace of the total spherical harmonic coefficient space. While IR performed similarly to SIGTT in the chosen *q* range, a rank-2 tensor cannot contain more than one local maximum per hemisphere. This poses a problem for the method in the case of the reciprocal-space map of the collagen meridional peak *q* range of bone, which contains two distinct maxima – an equatorial maximum from diffuse mineral scattering which lies along a great circle, and a meridional maximum from the spacing of the collagen fibril *d* spacing, which lies on the poles orthogonal to that great circle (*e.g.* Zhou *et al.*, 2016 ▸). This symmetry, which requires at least a rank-4 tensor, can be represented by both SIGTT and ZH (Guizar-Sicairos *et al.*, 2020 ▸).

## 3. Discussion

This work has demonstrated the SIGTT method for SAXSTT reconstruction of the reciprocal-space map in samples using a band-limited spherical function expressed in spherical harmonics (see Section 4 for details). In three case studies using simulated data with approximately zonally symmetric reciprocal-space maps, rank-2 tensors and complicated-textured higher-order reciprocal-space maps, the method produces results superior to the approaches of Liebi *et al.* (2018 ▸) and Gao *et al.* (2019 ▸). In addition, SIGTT is faster than the existing implementations of the methods of Liebi *et al.* (2018 ▸) and Gao *et al.* (2019 ▸), in many cases considerably so. SIGTT has also been shown to be robust to perturbations in the initial conditions when reconstructing experimental data. The reconstruction of the reciprocal-space map using higher spherical harmonic orders will enable the use of more specific methods of characterization that reveal information about the nature of the sample beyond the main orientation or adherence to a specific, predetermined symmetry. This representation of the reciprocal-space map in SIGTT makes it possible to study not just individual domains, but also the boundaries and transitions between them, including voxels with more than one orientation, which frequently occur in samples in the fields of biology and materials science (*e.g.* Georgiadis *et al.*, 2023 ▸; Maciel *et al.*, 2018 ▸). SIGTT can be applied to more complicated reciprocal-space maps, such as those that occur in SAXS measurements of samples with hexagonal symmetries [as done by Smarsly *et al.* (2005 ▸)], or in wide-angle X-ray scattering measurements. Wide-angle X-ray scattering measurements can be used by themselves or as a complement to SAXS measurements; see Mao *et al.* (2018 ▸) for an example of using small- and wide-angle X-ray scattering in combination for the study of a polymer under deformation. The extension of SAXSTT methods to also encompass wide-angle scattering is therefore a promising area of further study. Because it is not restricted to lower-order spherical harmonics, SIGTT is able to model reciprocal-space maps which are not well approximated by a rank-2 tensor, a case discussed by Georgiadis *et al.* (2021 ▸). For the same reasons, SIGTT should make it easier to reconstruct samples with smaller or less well organized domains [as done by Georgiadis *et al.* (2020 ▸)], as these would contain more transitory regions and a greater lack of symmetry in the reciprocal-space map. In this way, the reconstruction of the reciprocal-space map using band-limited spherical functions makes full use of the data obtained from the collection of scanning SAXS data at multiple angles, and opens up many new avenues of analysis. Finally, the anchoring of SIGTT in a framework of integral geometry and linear algebra highlights the potential for algorithms employing alternative schemes for data acquisition, optimization and representation of the reciprocal-space map, *e.g.* in the vein of the work of Sharma *et al.* (2017 ▸) on acquisition schemes for dark-field tomography, and that of Aslan *et al.* (2019 ▸) on ptycho-tomographic reconstruction.

## 4. Methods

### 4.1. Discretized formalism

The inverse problem to the forward model of equation (5) is to obtain the distributions 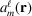 for a set of measured data.

In practical terms, the solution to the inverse problem is best discussed using a discrete formalism. Prior to reconstruction, measurements at a particular value of **q** are reduced by binning the measured pixel intensities into azimuthal segments, which corresponds to an integral over a great circle segment on the reciprocal-space sphere. Consequently, for the bin *i*, centered on φ_*i*_ of width Δφ, the spherical harmonics in equation (5) are integrated to give the coefficients for these bins, 

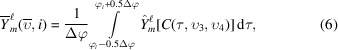

with υ_*i*_ as in equation (3), where the integration variable τ replaces the detector angle φ used in equation (5). Since this effectively blurs the reciprocal-space map, it constrains the maximum frequency that can be uniquely represented in the reduced data. It can be concluded that the ℓ_max_ of the fitted spherical function should follow 

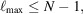

where *N* is the number of azimuthal bins for φ_*i*_ ∈ [0, π), due to the assumption of Friedel symmetry. This can be shown explicitly by expanding the *N* reciprocal-space map binned into azimuthal segments as a trigonometric polynomial,

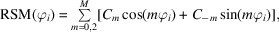

where *m* is the frequency of each component of the polynomial, and odd frequencies vanish due to Friedel symmetry. We observe that this has a unique solution only for *M* = *N* − 1 as a direct consequence of the Nyquist–Shannon sampling theorem (Shannon, 1949 ▸). It can be seen that a real trigonometric polynomial is isomorphic to a great circle cut of a spherical harmonic representation by noting that it is the expression for the azimuthal component of the real spherical harmonics (see Note 2 in the supporting information for details). The spherical harmonic rotation theorem implies that any great circle cut of a function expressed in spherical harmonics can be represented in a coordinate system where this great circle cut is the equator of the unit sphere. Since the polar angle is constant at the equator, this means that the great circle cut is determined only by the azimuthal component; thus, for any great circle cut of a spherical harmonic representation, there exists an equivalent trigonometric polynomial. Therefore by setting ℓ_max_ = *N* − 1 for the spherical harmonic representation, a unique solution exists for the great circle cut visible in each measurement. Consequently, the gradient contribution of that measurement becomes unique when solving the system (see Note 5 in the supporting information for details).

The sample volume, spanned by **r** in equation (2), is divided into cubic voxels, of the same size as the step size in the scanning SAXS measurement.

To pose our problem in matrix form, we describe our discretized system as a matrix **X** of *N* rows and *M* columns, where each row corresponds to a voxel, and each column corresponds to a spherical harmonic coefficient. Similarly, the measured data are described by an *I* × *J* matrix which we label **D**, with *I* being the number of scanned points and *J* the number of detector azimuthal segments across all rotation and tilt configurations. For simplicity of representation, we consider scans and detector azimuthal segments at different rotations and tilts to be distinct, so that **D** takes a sparse block-matrix form. The discrete equivalent of the projection operation 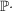 [see equation (4)], considered across all measured projections, is then given by a sparse *I* × *N* matrix **P**, describing a mapping between weighted sums of the *N* voxels to the *I* scanned points of the sample. Finally, the mapping from the spherical harmonic representation of the reciprocal-space map to the azimuthal segment-wise detector data is given by an *M* × *J* matrix **Y**, consisting of the *M* coefficients calculated in equation (6) for each of the *J* detector azimuthal segments.

This gives us the system of equations 

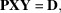

and we write the solution as the optimization of the least-squares problem 

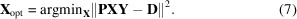

See Note 5 in the supporting information for details on how to solve this system with an iterative algorithm.

### 4.2. Reciprocal-space map evaluations

In tomography, the solution is generally assumed to be a reasonably smooth function, and we impose a regularization term on equation (7) to ensure this (Natterer & Wübbeling, 2001 ▸). Since we do not want the evaluation or comparison of reciprocal-space maps [defined by equations (1), (2)] to depend on our choice of coordinate system, it is necessary to use rotational invariants. A general rotational invariant is the canonical inner product of the spherical harmonics, known as the cross-spectrum function, 

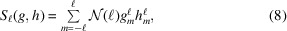

where 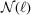 is a normalization factor that depends on the choice of spherical harmonic representation, ℓ is the cross-spectrum order, and *g* and *h* are two spherical functions (Wieczorek & Meschede, 2018 ▸). 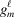 and 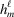 are the coefficients of the spherical harmonic representation of *g* and *h*, given by 

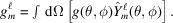




This discussion is therefore applicable to the analysis of spherical functions of any type, but in the particular case of SAXSTT, *f* and *g* are reciprocal-space maps as defined by equations (1), (2). To regularize the problem by imposing a smoothness condition, we compute a nearest-neighbor similarity term, 

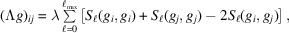

where the set of all (*i*, *j*) indicates neighboring pairs of voxels, *g*_*i*_ is the spherical function associated with each voxel and λ is a regularization coefficient.

In spherical harmonic coefficient space, this reduces to the squared discrete Laplacian operator on our system matrix weighted by λ, 

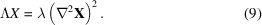

Minimizing this term results in maximizing the covariance between neighboring voxels, since 

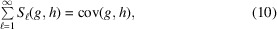

with *S*_ℓ_(*g*, *h*) defined in equation (8), and therefore we also have 

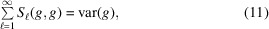

where, in particular, we refer in this work to var(*g*) as the anisotropic power of the reciprocal-space map represented by *g*.

Thus, with the addition of the regularization term in equation (9), the solution becomes 

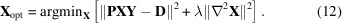

We define the isotropic component of a reciprocal-space map as its spherical mean 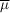 , and in spherical harmonic representation, 

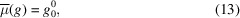

where *g* is the spherical function representing the reciprocal-space map.

We also define the relative anisotropy of a reciprocal-space map as 

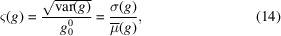

or, in other words, the standard deviation σ(*g*) of the spherical function *g* normalized by the spherical mean 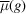 . We prefer to normalize by the mean rather than by the root-mean-square, because normalization by the root-mean-square would result in an upper bound of 1, which makes the contrast worse for highly anisotropic samples. This is useful to indicate the texture of a sample in many-voxel visualizations, and is used to color the glyphs in Fig. 4 ▸.

The relative anisotropy is comparable with the definition of the degree of orientation given by Bunk *et al.* (2009 ▸) as the ratio of the first Fourier component of an azimuthally integrated scattering pattern to the zeroth Fourier component (which is equal to the mean). This would correspond to calculating the relative anisotropy as defined by equation (14) using only the ℓ = 2 components of the spherical harmonic representation of the reciprocal-space map. We include coefficients beyond ℓ = 2, however, because we are interested in reciprocal-space maps of a more general class of symmetries, as well as figures of merit which are easily extended to other representations of the reciprocal-space map. The primary reason for not using a definition akin to that of the ρ parameter used by Fratzl *et al.* (2004 ▸), Pabisch *et al.* (2013 ▸), which uses the integrated peak intensity divided by the total intensity (peak intensity plus background intensity), is that defining the background intensity is difficult in a spherical harmonic representation. Moreover, using the standard deviation also incorporates information about the sharpness of the peaks, which is especially useful in cases where the background may be very small. The definition of the relative anisotropy in equation (14) is similar but not identical to the quantity also referred to as the degree of orientation by Liebi *et al.* (2018 ▸), which evaluates to the variance of the square root of the reconstructed reciprocal-space map divided by its mean.

### 4.3. Correlation calculations

Because the variances and covariances of reciprocal-space maps [equations (10), (11)] are rotational invariants, compositions of them are also invariants, and in particular 

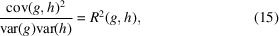

where var(*g*) is the variance of the spherical function *g*, cov(*g*, *h*) is the covariance of two spherical functions, and *R* ^2^(*g*, *h*) is the squared Pearson correlation coefficient of the two functions *g*, *h*. The squared Pearson correlation coefficient is used in the comparison of reconstructed reciprocal-space maps with those in simulated samples. The Pearson correlation coefficient does not map directly to other measures of similarity such as the difference in orientation; it is instead a summary metric of how similar two distributions are, up to a constant offset and a scaling factor. If *R* ^2^ is close to 0, then the distributions are very dissimilar, although it is still possible for other measures to show similarity of specific features, such as the orientation. If *R* ^2^ is close to 1, then the distributions are very similar and other measures will also show similarity (up to the possible offset of a constant and scaling factor); this generality is the reason why this is our statistic of choice. In the calculation of *R* ^2^ shown in Figs. 2 ▸, 5 ▸ and 6 ▸, the calculation is done between the reciprocal-space maps of each voxel in the simulated model (excluding empty voxels) and the reciprocal-space map of the same voxel in the reconstruction. The box plots in Figs. 2 ▸(*a*), 5 ▸(*a*) and 6 ▸(*a*) follow the original definitions of Tukey (1977 ▸). They are defined such that the colored rectangles span the interquartile range of the correlation distribution. The black ‘whiskers’ outside the colored rectangles span the smallest and largest value in the range [*Q*_1_ − 1.25 × (*Q*_3_ − *Q*_1_), *Q*_3_ + 1.25 × (*Q*_3_ − *Q*_1_)], where *Q*_*i*_ is the *i*th quartile of the distribution of *R* ^2^. Values outside the range of the ‘whiskers’ are represented by small circles, with each circle showing the mean *R* ^2^ of 100 reciprocal-space maps. If there is at least one, but fewer than 100 reciprocal-space maps above or below each whisker, a single black circle is shown, representing the mean of *R* ^2^ across these reciprocal-space maps. The median, equivalent to *Q*_2_, is shown by the colored markers in each box plot.

### 4.4. Simulation framework

For each of the three sample volumes, source points were determined such that the distance between each point was maximized: four source points for ‘M’, two source points for ‘T’ and five source points for ‘mammoth’. Band-limited spherical functions were constructed such that the spectral power of each order followed power-law decays with respect to ℓ, and assigned to each source point. The interior distance from each source to every other point in the volume was then approximately computed using a combination of a k-d tree and Dijkstra’s algorithm. The sources were assigned correlation lengths, with the assumption that for each order of each spherical function in the sample, correlation with the source would decay with distance like a Gaussian distribution with the correlation length as its standard deviation. The remaining spherical functions were then solved for under several constraints – in all cases, it was assumed that the spherical functions of neighboring voxels in the volume would be correlated with each other, and that the power of each order of the function in each voxel would equal a distance-weighted average of the power of its source. Moreover, it was required that all functions be non-negative. Non-negativity is difficult to enforce perfectly for spherical polynomials, but a dense sampling of each function was performed and the isotropic component was increased to eliminate all the detected negative points.

**Figure 9:**
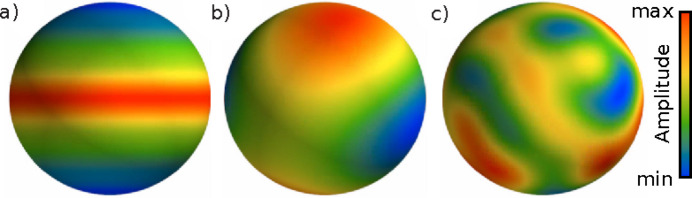
Reciprocal-space map symmetries for simulated samples. (*a*) Zonally symmetric spherical function with ℓ_max_ = 12 with its maximum around a great circle and a minimum at the poles, similar to the reciprocal-space maps of sample ‘M’. (*b*) Spherical function with ℓ_max_ = 2, similar to the reciprocal-space maps of sample ‘T’. (*c*) Spherical functions with ℓ_max_ = 8, with no particular symmetry constraints, similar to the reciprocal-space maps of sample ‘mammoth’. The color of the surface of each spherical function indicates its amplitude on a linear scale, scaled according to the largest and smallest value of each spherical function.

‘M’ consists of spherical polynomials up to ℓ_max_ = 12 that approximately follow zonal symmetry, *i.e.* there is for each spherical function an axis of orientation, about which they have approximate rotational symmetry. They can thus be well represented in a rotated spherical harmonic expansion with only *m* = 0 coefficients [see Fig. 9 ▸(*a*)]. To enforce zonal symmetry, each spherical function was required to correlate with the ℓ-weighted spherical harmonic Dirac δ function, 

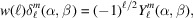

with (α, β) given by the fiber-like orientation vector of the ℓ = 2 component of the reciprocal-space map (see Note 3 in the supporting information for details on orientation analysis), and *w*(ℓ) being an ℓ-weighting function. In general, the condition of zonal symmetry requires compromise with the demand of continuity [enforced by minimizing the spherical harmonic Laplacian, as in equation (9)]. This is because the different orders of spherical harmonics have differing symmetries with respect to rotations. In effect, this leads to some attenuation of parts of the great circle of intensity. Hence, the reciprocal-space maps of ‘M’ are said to only approximately follow zonal symmetry. ‘T’ is entirely represented by symmetric rank-2 tensors, with a distribution required to be continuous [see Fig. 9 ▸(*b*)]. ‘Mammoth’ is represented by spherical polynomials with ℓ_max_ = 8, which in addition to being continuous have a damped ℓ = 2 component, such that the ℓ = 2 and ℓ = 4 components have approximately the same spectral power [see Fig. 9 ▸(*c*)]. This was done to approximate the type of spectral power distributions that occur in reciprocal-space maps which do not either have equatorial symmetry [with maxima concentrated around a great circle, akin to Fig. 9 ▸(*a*)] or meridional symmetry (with the maxima at the poles), such as in the region of the collagen meridional peak in bone (*e.g.* Zhou *et al.*, 2016 ▸), where the ℓ = 2 contributions from the meridional peak and the equatorial diffuse mineral scattering have opposing signs and thus tend to cancel out. The reciprocal-space map cuts generated from the projections of each simulation were divided into eight azimuthal segments, accounting for Friedel symmetry, and integrated over, emulating the azimuthal integration approach used for experimental data. The choice of eight bins was made based on previous usage in experimental data, as well as the fact that it will restrict the band-limit of spherical functions that can be precisely retrieved to ℓ_max_ = 6, meaning that it will not be possible for SIGTT to exactly solve for the reciprocal-space maps of the samples ‘M’ (ℓ_max_ = 12) and ‘mammoth’ (ℓ_max_ = 8).

### 4.5. Ensemble reconstructions

For the ensemble reconstructions shown in Fig. 8 ▸, each of the methods had their initial conditions randomized. In the case of SIGTT and IR, this consisted of randomizing the coefficients of their solution vectors at values several orders of magnitude below what is expected from their reconstructed value. In the case of ZH, the randomization was only applied to the Euler angles of the orientations of each voxel, which must be initialized before each reconstruction. The Euler angles were randomized such that the orientation vectors of each voxel were uniformly distributed on the unit sphere. The stepwise reconstruction procedure of Liebi *et al.* (2018 ▸) was then followed. In order to average over the result of the ensemble, the squared coefficients of the ZH reconstruction, performed with ℓ_max_ = 6, were expanded through Driscoll–Healy quadrature in a non-squared spherical harmonic representation up to ℓ_max_ = 12 (Driscoll & Healy, 1994 ▸). Tests incorporating higher orders and denser grids showed that this approach was accurate to a relative error of approximately 0.1% in the variance of the reciprocal-space map, which was deemed sufficient for the purpose of examining the reconstruction’s consistency across the ensemble. To evaluate the robustness of the reconstructions with respect to initial conditions, we defined an anisotropic power quotient for the reciprocal-space map in each voxel, 

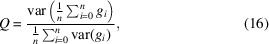

where *g*_*i*_ is the spherical function representing the reciprocal-space map in each voxel for reconstruction run *i*, and var(*g_i_*) is the anisotropic power of the reciprocal-space map as defined in equation (11), and *n* is the total number of reconstructions in the ensemble. We used *n* = 10, as this proved sufficient to illustrate the difference between the methods. If the reciprocal-space maps of every reconstruction in the ensemble are identical, the value of *Q* will be 1, and it will be in the range [0, 1) if the reciprocal-space maps differ.

### 4.6. Implementations

SIGTT, and the simulations used in this work, was implemented in Python. The most demanding parts of the code, projections and back-projections, are carried out using *Numba*, part of the software package *Mumott*, whereas other calculations are carried out in *NumPy* and *SciPy* (Harris *et al.*, 2020 ▸; Virtanen *et al.*, 2020 ▸; Lam *et al.*, 2015 ▸). The version of *Mumott* used in this work is available at https://doi.org/10.5281/zenodo.7798530. The most recent version of *Mumott* is made available at https://doi.org/10.5281/zenodo.7919448. The projection code, written specifically for this work, uses only CPU resources. It performs the John transform by using vectors to trace out the lines of integration, and sampling the voxels that these intersect with, in proportion to the lengths of the intersecting segments. Visualizations of the three-dimensional reconstructions were created using the Python package *Mayavi* (Ramachandran & Varoquaux, 2011 ▸). The color maps used throughout this paper were generated with the help of *ColorCET* (https://colorcet.com) (Kovesi, 2015 ▸). All computations were performed on a workstation using a 12-core, 4.6 GHz AMD Ryzen 9 3900X CPU and 64 GB DDR4 2666 MHz RAM. For the IR and ZH methods, the original code from the *cSAXS* software package written in Matlab was used, with modifications for optimization and termination of each reconstruction upon convergence. The projection code in this package samples voxels using coordinate transforms and bilinear interpolation. It slices the sample along the plane of integration, and samples the four voxels closest to the line of integration, based on the distance in the plane of projection. Because it effectively treats the projection of each voxel as a square at every angle of projection, and does not consider the full three-dimensional distance between voxels, this approach suffers from high-frequency artifacts. However, following testing, this approach was deemed sufficiently accurate for the purpose of comparing ZH and IR with SIGTT.

## 5. Data availability

The simulated data created for and used in this work are available at https://doi.org/10.5281/zenodo.7673985. A notebook demonstrating the analysis and reconstruction using *Mumott* is available at https://doi.org/10.5281/zenodo.7799517.

## Supplementary Material

Supporting information. DOI: 10.1107/S205327332300863X/ae5133sup1.pdf
