# Supplementary material for: Small-angle scattering tensor tomography algorithm for robust reconstruction of complex textures
Source: Acta Crystallogr A Found Adv. 2023 Oct 19;79(Pt 6):515–26. doi: 10.1107/S205327332300863X (PMC10626654; doi:10.1107/S205327332300863X)
Supplement: Supplementary file 1 [file a-79-00515-sup1.pdf]

FOUNDATIONS
ADVANCES

Volume 79 (2023)

Supporting information for article:

Small-angle scattering tensor tomography algorithm for robust reconstruction of complex textures

Leonard C. Nielsen, Paul Erhart, Manuel Guizar-Sicairos and Marianne Liebi

Supplementary Note 1: Coordinate mapping

Supplementary Note 2: Tensors and spherical harmonics

Supplementary Note 3: Orientation analysis

Supplementary Note 4: Additional results

Supplementary Note 5: Solving regularized least-squares system

Supplementary Note 6: Implementation details

Supplementary Note 1. COORDINATE MAPPING

In the "Formalism" section of the paper, the John Transform is described using vectors in the local coordinate system of the sample. This is the most convenient way to parameterize the system, since it is the sample volume that is ultimately being solved for. However, for practical reasons, it is useful to lay out how one can map a coordinate system of a laboratory frame, relative to which the sample is moving. In general, we can specify the relationship between the laboratory system and the sample system by choosing a set of vectors and rotation axes at zero tilt and rotation, and then applying any rotations of the sample to those vectors. Three positioning vectors are needed - one for the direction of the x-ray beam, and two which indicate how the beam moves relative to the sample during scanning. We may call these $\mathbf{p}$, $\mathbf{w}_j$ and $\mathbf{w}_k$. The vectors $\mathbf{w}_j$ and $\mathbf{w}_k$ should be shifted to align the system, such that if $\mathbf{w}_j = \mathbf{w}_k = 0$, the beam intersects the center of the sample at all rotations. Additionally, two are required to indicate how angles on the detector map to the reciprocal space sphere, we may call these $\mathbf{q}_0$ and $\mathbf{q}_{90}$, defined to indicate where on the reciprocal space sphere detector angle 0 and 90 lie. Two orthogonal rotation axes need to be defined, orthogonal to the projection direction, in order to translate rotations to the sphere of projection, which we call $\hat{\alpha}$ and $\hat{\beta}$. These do not necessarily have to correspond to the axes of real rotation stages. Then, any rotation $\mathbf{R}$ that the sample is subject to must be decomposed into three components, $\mathbf{R}_\alpha(\alpha)$, $\mathbf{R}_\beta(\beta)$, and $\mathbf{R}_p(\gamma)$, for rotations around $\hat{\alpha}$, $\hat{\beta}$ and $\mathbf{p}$ respectively, such that

$$\mathbf{R} = \mathbf{R}_p(\gamma)\mathbf{R}_\beta(\beta)\mathbf{R}_\alpha(\alpha)$$

Then, any one of the vectors $\mathbf{a}$ in the zero-rotation system will become

$$\mathbf{a}' = \mathbf{R}_\alpha^T(\alpha)\mathbf{R}_\beta^T(\beta)\mathbf{R}_p^T(\gamma)\mathbf{a}$$

The John transform parameters j and k are then given by the norms of $\mathbf{w}_j$ and $\mathbf{w}_k$, whereas α and β are the arguments of the rotation matrices $\mathbf{R}_\alpha$ and $\mathbf{R}_\beta$. The rotation matrix $\mathbf{R}_p$ simply specifies a rotation around the axis of projection, and therefore only changes the mapping of j and k, rather than representing a degree of freedom on the sphere of projection. The reciprocal space angles θ and ϕ are given by the azimuthal and polar angle of the reciprocal space vector

$$\mathbf{q}' = \mathbf{R}^T(\cos(\varphi)\mathbf{q}_0 + \sin(\varphi)\mathbf{q}_{90})$$

where φ is the angle on the detector.

The vectors $\mathbf{v}$ and $\mathbf{u}$ in the John transform are given by

$$\mathbf{v} = \mathbf{R}^T(\mathbf{w_j} + \mathbf{w_k})$$
$$\mathbf{u} = \mathbf{R}^T(\mathbf{p})$$

Using these rules, one can calculate the John transform using rotation information from any laboratory coordinate system.

Supplementary Note 2. TENSORS AND SPHERICAL HARMONICS

The comparison between methods using spherical harmonic representations and tensor representation merits a brief discussion of isomorphisms between these parameterizations. Spherical harmonics are simply harmonic polynomials on the sphere. In this work we use the *real* spherical harmonics, which we may define as

$$\hat{Y}^{\ell}_{m}(\theta,\phi) = \begin{cases} L^{\ell}_{m}(\theta)\cos(m\phi) & m \geq 0 \\ L^{\ell}_{m}(\theta)\sin(|m|\phi) & m < 0 \end{cases}$$

where θ is the polar angle, ϕ is the azimuthal angle, $L^{\ell}_{m}(\theta)$ is the associated Legendre polynomial of degree ℓ and order m, with $\ell \geq 0$ and $|m| \leq \ell$. Because of this, they have an intuitive mapping to traceless symmetric Cartesian tensors when representing functions on the unit sphere. In particular, a band-limited spherical function represented in $\ell = n$ maps directly to a traceless rank-n tensor. The rank-0 tensor is simply a constant, so it naturally maps to $\hat{Y}^{0}_{0}(\theta,\phi)$, which is a constant term. This mapping naturally leads to restricting the mapping to *traceless* tensors, because the trace of a tensor on the unit sphere is a constant term. Moreover, a spherical harmonic of $\ell = n$ can be represented as a polynomial of Cartesian coordinates of degree n, that is to say

$$\begin{aligned} P(n,\mathbf{x}) &= a_m \hat{Y}^{n}_{m}(\mathbf{x}) \\ x_i x_i &= 1 \end{aligned}$$

where Einstein summation over repeated indices is used, and $\mathbf{a}$ is some vector of $2n+1$ coefficients. In the same way, we have for a traceless rank-n tensor T the mapping

$$P(n,\mathbf{x}) = T^{ijk\cdots} x_i x_j x_k \ldots,$$

$P(n,\mathbf{x})$ is the value of some polynomial of degree n at $\mathbf{x}$. Restricting ourselves to the unit sphere, it is then the case that

$$a_m \hat{Y}^{n}_{m}(\mathbf{x}) = T^{ijk\cdots} x_i x_j x_k \ldots,$$

By the additivity of polynomials, this mapping can be extended to sums of traceless symmetric tensors tensors of arbitrary orders, and through Gaussian elimination, it is straightforward to calculate a mapping between spherical harmonics and symmetric Cartesian tensors. Tensor and spherical harmonic representations of functions on the unit sphere are thus isomorphic, and their different analytical and algebraic properties render them suitable for different purposes. Spherical harmonics are especially suitable for calculations where rotational invariance is crucial, and for calculating spherical statistics such as the variance or mean over the sphere. On the other hand, it would be easier to perform tensor calculus (such as computing the divergence or curl) on Cartesian tensors.

Supplementary Note 3. ORIENTATION ANALYSIS

Orientation analysis is used in figures 4 and 6 of the main work, as well as in the creation of the simulated sample "M". The identification of a reciprocal space map's orientation is done by eigenvector-eigenvalue decomposition of its $\ell = 2$ spherical harmonic coefficients. The $\ell = 2$ coefficients are translated into a symmetric traceless rank-2 tensor by solving for each coefficient in the tensor's polynomial representation. Specifically, on the unit sphere, the two representations may be expanded as

$$\begin{bmatrix} x & y & z \end{bmatrix} \begin{bmatrix} T_{xx} & T_{xy} & T_{xz} \\ T_{xy} & T_{yy} & T_{yz} \\ T_{xz} & T_{zy} & T_{zz} \end{bmatrix} \begin{bmatrix} x \\ y \\ z \end{bmatrix} = x^2 T_{xx} + y^2 T_{yy} + z^2 T_{zz} + 2(xyT_{xy} + xzT_{xz} + yzT_{yz})$$

$$\mathcal{N}(2) \begin{bmatrix} a^2_0 & a^2_1 & a^2_{-1} & a^2_2 & a^2_{-2} \end{bmatrix} \begin{bmatrix} \frac{2z^2-y^2-x^2}{2\sqrt{3}} \\ xz \\ yz \\ xy \\ \frac{x^2-y^2}{2} \end{bmatrix} = \mathcal{N}(2)(x^2(\frac{a^2_{-2}}{2} - \frac{a^2_0}{2\sqrt{3}}) + y^2(-\frac{a^2_{-2}}{2} - \frac{a^2_0}{2\sqrt{3}}) + z^2\frac{a^2_0}{\sqrt{3}} + a^2_2 xy + a^2_1 xz + a^2_{-1} yz)$$

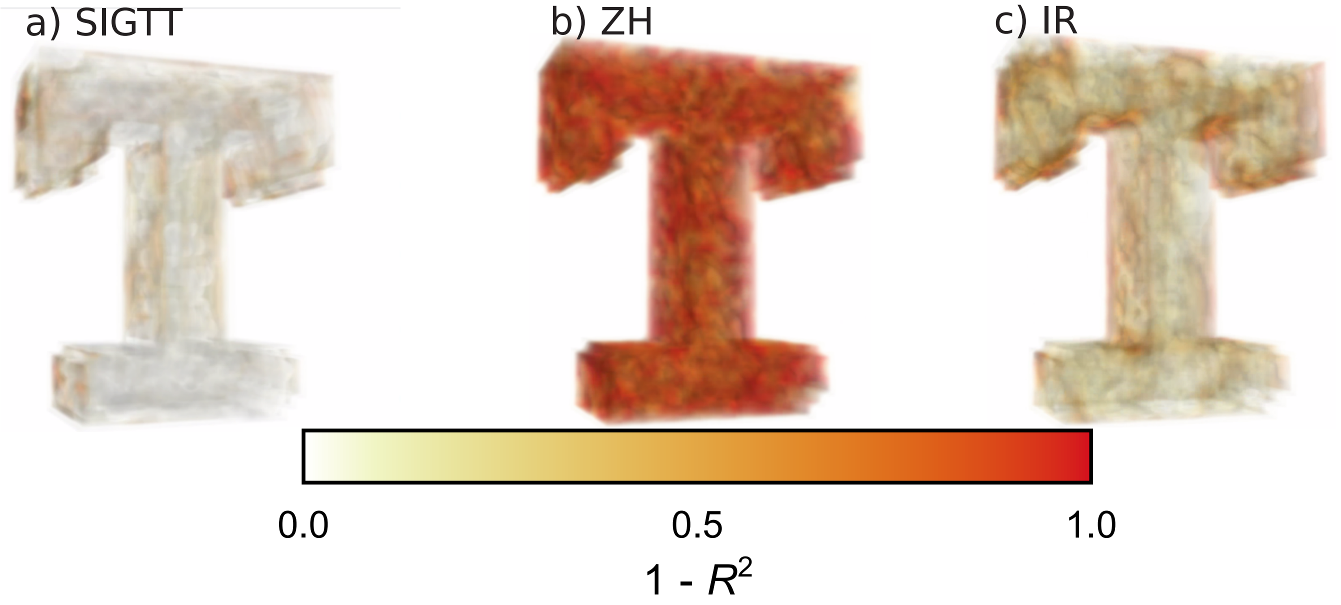

Supplementary Figure S-1. **Errors for "T".** Volume renders of the errors of a) SIGTT, b) SH, and c) IR for sample "T", defined as $1 - R^2$, where R^2 is given by equation (15) of the main text. Larger errors are rendered with greater opacity and are thus visible even if they are in the interior.

where $\mathcal{N}(2)$ is a normalization factor depending on the spherical harmonic representation, a^2_m is a spherical harmonic coefficient with $\ell = 2$, T_{ii} are components of a rank-2 tensor, and (x, y, z) are cartesian coordinates. By equating these two systems of equations, we can then identify each T_{ii} with a linear combination of coefficients a^2_m and thus construct a rank-2 tensor out of spherical harmonic coefficients. Subsequently, we may solve the eigenvalue problem for the rank-2 tensor, to obtain three eigenvalues (which will sum to zero as the tensor will be traceless) and three orthogonal eigenvectors. For a rank-2 tensor with three distinct eigenvalues, the eigenvectors associated with the largest and smallest eigenvalues correspond to the location of the minima and maxima of the spherical function. The eigenvector of the central eigenvalue corresponds to a saddle point. If there is degeneracy in the eigenvalues, but they are nonzero, the unique eigenvalue corresponds to one extremum (minimum if it is negative, maximum if it is positive), whereas the two identical eigenvalues correspond to two orthogonal points on a set of extreme points which lie along a great circle. The orientation analysis then depends on assumptions about the symmetry of the nanostructure associated with the reciprocal space map. If the nanostructure is known or assumed to be scattering from fiber-like structures, orthogonal to the direction of the fibers, the eigenvector associated with the smallest eigenvalue is taken to define the orientation. On the other hand, if the reciprocal space map is known or assumed to be scattering along the orientation, the eigenvector associated with the largest eigenvalue is taken to define the orientation. Nanostructures which contain both of these scattering tendencies, such as bone around the q-range of the collagen peak, cannot in general be robustly analyzed in this manner, because each scattering tendency will tend to cancel out the contribution from the other to the rank-2 tensor component of the reciprocal space map. Such reciprocal space maps require a different treatment, such as correlation analysis with an ensemble of model functions.

Supplementary Note 4. ADDITIONAL RESULTS

This section shows some additional results for the simulation comparisons to supplement those in the main work.

Volume renders of the errors of each method for "T" can be seen in Supplementary Figure **S-1**. It is evident from the figure that Spherical Integral Geometric Tensor Tomography (SIGTT) has the smallest errors, and tha the large errors are concentrated around the edges. It is followed by IR, which has large regions of small errors but larger errors around the same regions as SIGTT However, the errors for SH are far greater than for either of the other two methods. This is partly because the squared polynomials of SH cannot exactly represent rank-2 tensors, and partly because sample "T" is not constrained to zonal symmetry.

A similar set of volume renders of errors for each method for "mammoth" can be seen in Supplementary Figure **S-2**. Again, SIGTT has the smallest errors, with larger errors around some edge regions. In this case, SH and IR are more similar, having large errors throughout, but IR consistently appears to have somewhat smaller errors. This is

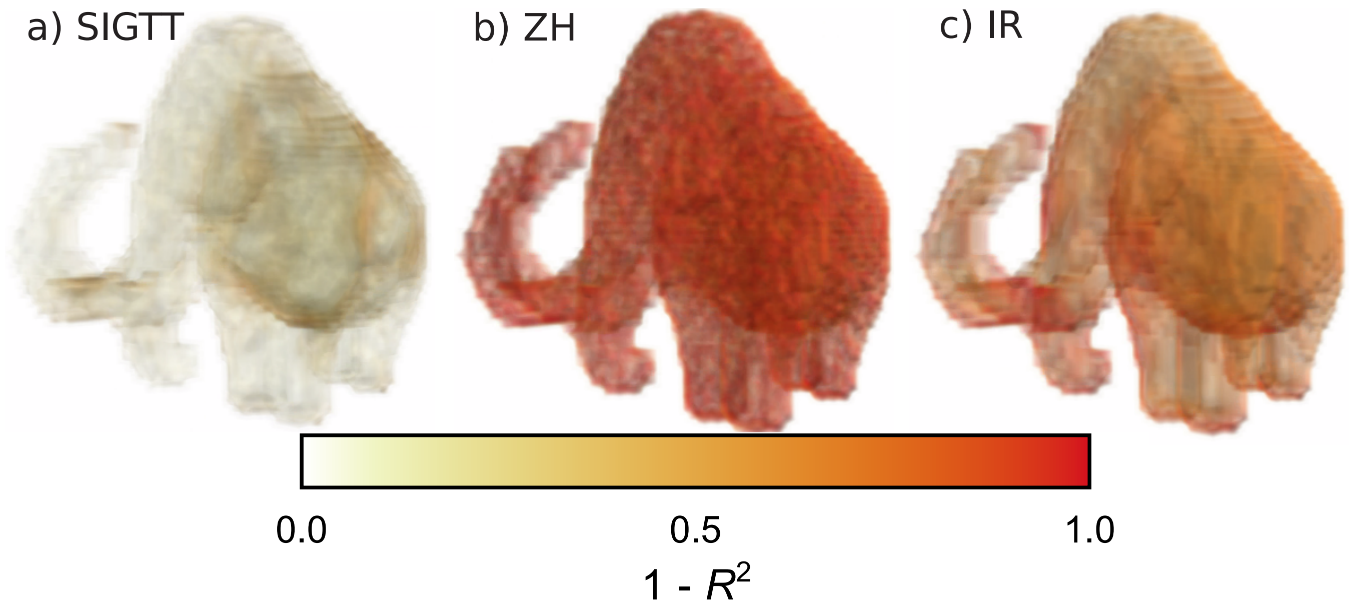

Supplementary Figure S-2. **Errors for "mammoth".** Volume renders of the errors of of a) SIGTT, b) SH, and c) IR for sample "mammoth", defined as $1 - R^2$, where R^2 is given by equation (15) of the main text. Larger errors are rendered with greater opacity and are thus visible even if they are in the interior.

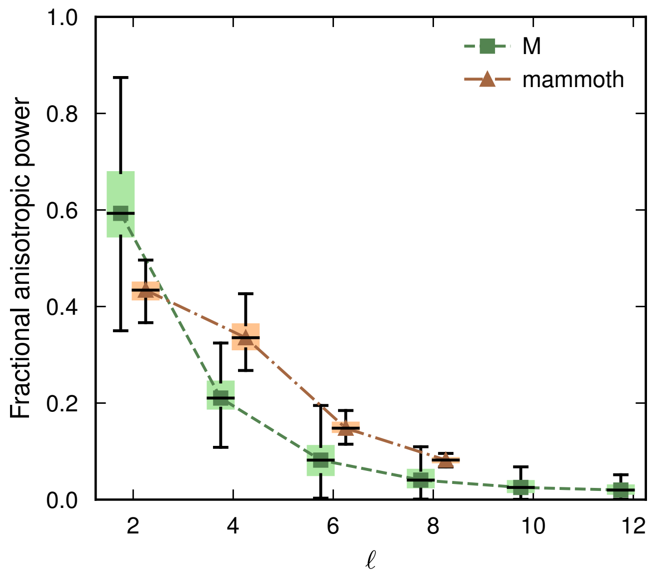

Supplementary Figure S-3. **Fractional anisotropic power.** Distribution of the fraction of anisotropic power (equation (S1)) at each ℓ for the samples "M" and "mammoth". Sample "M" has a distribution that is more dominated by the $\ell = 2$ component, and a steeper decline, whereas for "mammoth", the $\ell = 2$ and $\ell = 4$ components are closer to each other, and $\ell = 6$ and $\ell = 8$ also occupy a larger fraction than for "M".

consistent with figure 5 in the main text. where is it clear that IR can achieve a correlation around 0.3, but not more, whereas the correlation for SH is close to 0. These large errors for IR and SH are due to the fact that the "mammoth" sample has a small rank-2 tensor component on the one hand, and on the other hand, no zonal symmetry constraint.

To examine the distribution of anisotropic power in samples, we define the quantity

$$F_\ell(g) = \frac{S_\ell(g)}{\mathrm{var}(g)} \tag{S1}$$

where g is a spherical function, and S_ℓ and $\mathrm{var}(g)$ are defined in Equations (8) and (11) in the main text respectively. This quantity, referred to as the fractional anisotropic power, is then evaluated for every voxel that is part of the sample. In Supplementary Figure **S-3**, we see box plots of the fractional anisotropic power of "M" and "mammoth". While "M" has a spectrum that goes up to $\ell = 12$, the orders about $\ell = 6$ only contribute a small amount of the overall power.

Supplementary Note 5. SOLVING REGULARIZED LEAST-SQUARES SYSTEM

The system of linear equations to be solved in SIGTT is given as

$$\mathbf{X}_{\mathrm{opt}} = \underset{\mathbf{X}}{\mathrm{argmin}} \left[\|\mathbf{PXY} - \mathbf{D}\|^2 + \lambda \|\nabla^2 \mathbf{X}\|^2 \right].$$

This system is solved through the gradient-based method L-BFGS-B. In detail, a general solution using a quasi-Newton algorithm may be written

$$\begin{aligned} 2r_i &= \|\mathbf{P}\mathbf{X}_i\mathbf{Y} - \mathbf{D}\|^2 + \lambda\|\nabla^2\mathbf{X}_i\|^2 \\ \nabla r_i &= \mathbf{P}^T(\mathbf{P}\mathbf{X}_i\mathbf{Y} - \mathbf{D})\mathbf{Y}^T + \lambda\nabla^2\mathbf{X}_i \\ \mathbf{X}_{i+1} &= \mathbf{X}_i - \alpha_i\nabla r_i + \beta_i\mathbf{p}_i \end{aligned}$$

where r_i is the residual for iteration i, $\mathbf{X}_i$ is the estimated solution, α_i and β_i are method-dependent scalars, and $\mathbf{p}_i$ is a method-dependent momentum term. While the multiplication with $\mathbf{Y}^T$ is directly implemented as a matrix multiplication, this is not practical to do for the adjoint projection operation $\mathbf{P}^T$, due to the prohibitively large size of the matrix. Instead, it is computed using the common iterative tomographic method of back-projection of the residual.

Supplementary Note 6. IMPLEMENTATION DETAILS

For each of the simulated data sets, an overall signal-to-noise ratio (SNR) was estimated. Noise was added to each projected simulated data point per

$$I_{noise} = \frac{\mathrm{poisson}(I \cdot k)}{k}$$

with k being a noise parameter, and poisson(x) being a sampling of x with added Poisson noise. The values used for k were $k = 10^t$ with $t \in \{2, 1.5, 1, 0.5, 0\}$, This means that each data point will be a random variable with variance I/k. Thus, the signal-to-noise ratio for a simulated data set with noise parameter k was estimated as

$$\mathrm{SNR} \approx \sqrt{\overline{\mu}(I_i)k}$$

with $\overline{\mu}(I_i)$ being the average intensity of all non-background data points in that data set. This SNR should be understood principally as a relative measure for a particular simulation, not as an absolute measure.
